# Cardiovascular Anomalies Associated With Esophageal Atresia: A 23-Year Single-Center Experience

**DOI:** 10.7759/cureus.90463

**Published:** 2025-08-19

**Authors:** Saud AlJadaan, Nourah Alsabty, Fahad AlTurki, Rakan Aldusari, Abdullah Almusallam

**Affiliations:** 1 Pediatric Surgery, King Abdullah Specialized Children's Hospital, Riyadh, SAU; 2 Pediatric Surgery, King Saud University Medical City, Riyadh, SAU; 3 Pediatric Surgery, Diriyah Hospital, Riyadh, SAU; 4 General Surgery, King Saud Bin Abdulaziz University for Health Sciences College of Medicine, Riyadh, SAU

**Keywords:** congenital cardiovascular malformation, congenital heart defects, esophageal atresia, tracheoesophageal fistula, vactrel association

## Abstract

Background: Esophageal atresia (EA) is a rare congenital anomaly frequently associated with congenital heart disease (CHD). This study aimed to evaluate the incidence and characteristics of cardiac anomalies in EA patients treated at a tertiary center in Saudi Arabia.

Methods: A retrospective review was conducted at National Guard Health Affairs-Riyadh. Medical records of 87 patients diagnosed with EA between 2000 and 2023 were analyzed for EA type, syndromic associations, cardiac anomalies, and the need for cardiac interventions. Statistical analysis was performed using IBM SPSS Statistics for Windows, Version 26 (Released 2019; IBM Corp., Armonk, New York).

Results: Of the 87 patients, 75.9% had cardiac anomalies, which decreased to 42.5% when excluding patent ductus arteriosus (PDA) and patent foramen ovale (PFO). The most common EA type was Type C (80.5%). Syndromic diagnoses were identified in 17.3% of patients, with trisomy 21 being the most common. Cardiac anomalies were significantly associated with female gender (p = 0.013) and syndromic status (p = 0.007). Ventricular septal defect (VSD) and coarctation of the aorta were significantly more frequent in syndromic patients. Nine patients required cardiac intervention during the same admission. The need for cardiac intervention was significantly associated with syndromic status, suggesting that these patients may have more severe cardiac anomalies.

Conclusion: Cardiac anomalies excluding PDA and PFO occurred in 42.5% of EA patients. ASD and VSD were the most common findings. Syndromic and female patients had higher rates of cardiac anomalies and cardiac interventions. These results emphasize the need for thorough preoperative cardiac evaluation and multidisciplinary management in EA patients.

## Introduction

Esophageal atresia (EA) is a rare congenital anomaly with an estimated prevalence of 1.77 to 3.61 per 10,000 births [1]. The estimated prevalence of EA in Saudi Arabia is 3 per 10,000 births [2]. There are five types of EA: Type A is EA without a fistula, Type B is EA with a proximal tracheoesophageal fistula (TEF), Type C is EA with a distal TEF, Type D is EA with both proximal and distal TEF, and Type H is a TEF without EA [3]. In most cases, EA occurs sporadically and in non-syndromic patients [3,4]. However, the relationships between EA and syndromes have been well described [5]. The syndromes include trisomy 21, trisomy 18, and CHARGE (coloboma, heart defect, atresia choanae, retarded growth and development, genital abnormality, ear abnormality) syndrome [5]. VACTERL (vertebral defects, anal atresia, cardiac defects, tracheo-esophageal fistula, renal anomalies, and limb abnormalities) is the most common association with EA and includes vertebral defects, anal atresia, cardiac defects, TEF, renal anomalies, and limb abnormalities [6]. Congenital heart disease (CHD) is the most frequently co-occurring anomaly, affecting approximately 40% of EA patients [6]. Moreover, it has been suggested that CHD is 23 times more common in patients with EA [6]. Al-Salem et al. reported that among EA patients with or without TEF, CHD was the most common anomaly, occurring in 44.7% of patients [7]. Another study conducted locally by Rejjal et al. reported that the prevalence of CHD among EA/TEF patients was 19% [8]. The most common CHDs identified in that study included ventricular septal defect (VSD), atrial septal defect (ASD), patent ductus arteriosus (PDA), and dextrocardia. The presence of a cardiac anomaly is a main predictive factor for EA outcome [5]. Local studies on EA and its associated anomalies have been limited and have been conducted at widely varying time points. This study aims to examine the associated cardiac anomalies in EA patients and provide a comprehensive report on the incidence and characteristics of cardiac anomalies in EA/TEF patients, updating local literature with recent findings, especially given the advancements in neonatal care.

This article was previously presented as an oral presentation at the International Conference on Surgeries in Cardiology (ICSIC-2024), June 18, 2024.

## Materials and methods

This retrospective cross-sectional study was conducted at the National Guard Health Affairs-Riyadh (NGHA-R), a tertiary referral center that treats many patients annually. NGHA-R has a specialized pediatric surgery department, a dedicated children’s hospital, a large emergency department, an advanced neonatal intensive care unit (NICU), a dedicated cardiac center, and multiple other specialized departments. At this center, EA is diagnosed by confirming a coiled nasogastric tube (NGT) on a chest X-ray. Type H TEF is diagnosed using fluoroscopy and/or bronchoscopy. All patients diagnosed with EA undergo echocardiography (ECHO) before surgery as part of the preoperative workup. Chromosomal studies are performed when dysmorphic features are noted following assessment by the genetics team.

Purposive sampling was used to include the entire study population. Data were extracted from the electronic health records of the NGHA database (BESTCARE 2.0A), which was launched in 2015. Data for patients born before 2015 were retrieved from paper files. All patients diagnosed with EA at NGHA-R between January 2000 and December 2023 were included in the study, while patients who were operated on outside the institution were excluded to minimize missing data.

After receiving approval from the Institutional Review Board (IRB) of King Abdullah International Medical Research Center (approval No. IRB/1079/22), data collection began. A total of 87 patients met the inclusion criteria. Their names and MRNs were replaced with serial numbers to ensure privacy. An Excel sheet was used for data entry following manual extraction from EMR and paper files. The variables included gender, gestational age, EA type, aortic arch orientation, cardiac anomalies, syndromes, and the need for cardiac interventions. Gestational age was categorized as preterm (<37 weeks) or full-term (≥37 weeks) [9]. All variables were categorical.

The data were analyzed using the Statistical Package for IBM SPSS Statistics for Windows, Version 26 (Released 2019; IBM Corp., Armonk, New York). Categorical variables are described as percentages. Fisher’s exact test and chi-square tests were used for correlation analyses. A 95% confidence interval was calculated, and a p-value ≤ 0.05 was considered statistically significant.

## Results

A total of 87 pediatric patients were included. The baseline characteristics of the study population are presented in Table 1.

**Table 1:** Characteristics of esophageal atresia patients (n = 87). EA: esophageal atresia, TEF: tracheoesophageal fistula, CHARGE: coloboma, heart defect, atresia choanae, retarded growth and development, genital abnormality, ear abnormality.

Variable	Category	n (%)
Sex	Male	49 (56.3)
	Female	38 (43.7)
Gestational age	Full term	43 (49.4)
	Premature	44 (50.6)
Type of esophageal atresia	Esophageal atresia without TEF (type A)	14 (16.1)
	Esophageal atresia with distal TEF (type C)	70 (80.5)
	TEF without esophageal atresia (type H)	3 (3.4)
Orientation of the aortic arch	Left	86 (98.9)
	Right	1 (1.1)
Syndromic	No	72 (82.7)
	Yes	15 (17.3)
Type of syndrome (n = 15)	Trisomy 21	7 (46.66)
	Trisomy 18	4 (26.66)
	CHARGE syndrome	2 (13.33)
	Mucopolysaccharidosis type 4	1 (6.66)
	Fanconi anemia	1 (6.66)

Males accounted for 56.3% (n = 49) of the sample. Full-term patients accounted for 49.4% (n = 43) of the sample. The most common type of EA was EA with distal TEF (Type C) (80.5%, n = 70), followed by EA without TEF (16.1%, n = 14), and 3.4% (n = 3) had TEF without EA (Type H). Syndromic patients accounted for 17.3% of the study population, with trisomy 21 being the most common syndrome (46.6%), followed by trisomy 18 (26.6%). Two patients had CHARGE syndrome.

All patients underwent ECHO before surgery. Three-quarters of the patients had cardiac anomalies (75.9%), but when PDA and patent foramen ovale (PFO) were excluded, 42.5% of patients had cardiac anomalies. The incidence of cardiac anomalies is presented in Table 2.

**Table 2:** Cardiac anomalies among esophageal atresia patients (n = 87).

Cardiac Anomaly	Category	n (%)
Patent ductus arteriosus (PDA)	Yes	40 (45.9)
	No	47 (54.0)
Patent foramen ovale (PFO)	Yes	20 (22.9)
	No	67 (77.0)
Atrial septal defect (ASD)	Yes	23 (26.4)
	No	64 (73.5)
Ventricular septal defect (VSD)	Yes	19 (21.8)
	No	68 (78.1)
Tetralogy of fallot	Yes	3 (3.4)
	No	84 (96.5)
Coarctation of the aorta (COA)	Yes	2 (2.2)
	No	85 (97.7)
Common atrium	Yes	1 (1.1)
	No	86 (98.8)
Bicuspid aortic valve	Yes	1 (1.1)
	No	86 (98.8)
Bicuspid pulmonary valve	Yes	1 (1.1)
	No	86 (98.8)
Double outlet right ventricle	Yes	1 (1.1)
	No	86 (98.8)
Vascular ring	Yes	1 (1.1)
	No	86 (98.8)
Aortopulmonary collateral	Yes	1 (1.1)
	No	86 (98.8)
Hypoplastic left heart syndrome	Yes	1 (1.1)
	No	86 (98.8)
Aberrant origin of the right subclavian syndrome	Yes	1 (1.1)
	No	86 (98.8)
Dilated right sided heart	Yes	1 (1.1)
	No	86 (98.8)
Bronchopulmonary dysplasia	Yes	1 (1.1)
	No	86 (98.8)
Tortus, hypoplastic transverse aortic arch	Yes	1 (1.1)
	No	86 (98.8)
Truncus arteriosus	Yes	1 (1.1)
	No	86 (98.8)

The left aortic arch orientation was observed in all patients except one, who had a right aortic arch. Nine patients required cardiac intervention during the same admission, 5 of whom were syndromic patients (p = 0.010). Six patients underwent intervention after EA repair, one underwent vascular ring repair during EA repair, and two patients with EA without TEF required cardiac intervention before esophageal anastomosis. A significant correlation was observed between congenital cardiac anomalies (excluding PDA and PFO) and female gender (p = 0.013), as well as syndromic status (p = 0.007). Eleven of the syndromic patients were female (p = 0.040). Although the overall sample had a slight male predominance (56%), the syndromic patients were predominantly female (73%, p = 0.021). VSD (p = 0.020) and coarctation of the aorta (COA) (p = 0.033) were significantly associated with syndromic status. Two patients had COA, and both were syndromic (CHARGE syndrome and trisomy 18). No significant correlation was found between EA type or gestational age and cardiac anomalies.

## Discussion

The study’s aim was to investigate the prevalence and types of pediatric cardiac anomalies among patients with EA managed at King Abdulaziz Medical City (KAMC), examining a cohort of 87 patients with EA. The overall prevalence of cardiac anomalies in this population was 42.5%, within the previously reported regional range of 19% to 44% [7,8]. This variability in prevalence across studies may be attributed to several factors, including advancements in medical imaging techniques and inconsistencies in whether PDA and patent foramen ovale (PFO) were included in the classification of cardiac anomalies [7].

In our analysis, the prevalence of cardiac anomalies was calculated excluding PDA and PFO, as most patients undergo echocardiography within the first 48 hours of life, and it is well established that PDA and PFO often close spontaneously within this early neonatal period in healthy newborns [10]. ASDs and VSDs emerged as the most commonly identified cardiac anomalies, consistent with previous findings from other Saudi studies [7,8,11]. The prevalence of complex CHDs, including tetralogy of Fallot (TOF), common atrium, and bicuspid aortic valve, was low and showed no substantial change compared with earlier reports, including a notable study conducted in 1999 [8]. This may reflect an overall lower prevalence of such complex anomalies in the Saudi population [11]. Among the observed anomalies, the prevalence of a right-sided aortic arch was notably low at 1.1%, in contrast to the 9% reported in the literature [8].

While prematurity is recognized as a risk factor for congenital cardiac anomalies [12], no statistically significant correlation was observed in patients with EA. Syndromic patients accounted for 17.3%, higher than the 10% reported in the literature [13]. This difference could be attributed to the high rate of consanguinity in Saudi Arabia, which is observed in half of the population [14]. All reported syndromes and chromosomal anomalies in our sample have known associations in the literature with EA, except mucopolysaccharidosis 4A (Morquio syndrome) [15,16]. There was a higher proportion of female patients among the syndromic group, which may be explained by the known female predominance in individuals with trisomy 18 [17]. The need for cardiac intervention was significantly associated with syndromic patients, suggesting that they tend to present with more severe or complex cardiac anomalies.

While the findings of this research provide an overview of cardiac anomalies in EA patients, several limitations should be acknowledged. Premature infants were not subcategorized based on the degree of prematurity, which may have affected the ability to detect a correlation between prematurity and cardiac anomalies. Another limitation is that KAMC is a major referral center with a cardiac center, which may have led to an overestimation of the number of syndromic and complex cardiac patients. Lastly, the retrospective nature of the study and the small sample size may limit the generalizability of the findings.

## Conclusions

This study revealed that 42.5% of EA patients (excluding those with PDA and PFO) had cardiac anomalies. ASD and VSD were the most common cardiac anomalies. The incidence of TOF and other complex cardiac anomalies was low. Female patients and syndromic patients had a greater risk of cardiac anomalies and required more cardiac interventions. Further research is needed to determine the underlying causes of these associations.
